# Supplementary material for: Distinct structural features of Pseudomonas aeruginosa ATP synthase revealed by cryo-electron microscopy
Source: Nat Commun. 2025 Dec 9;17:406. doi: 10.1038/s41467-025-67100-0 (PMC12796488; doi:10.1038/s41467-025-67100-0)
Supplement: Supplementary file 1 — Supplementary Information [file 41467_2025_67100_MOESM1_ESM.pdf]

SUPPLEMENTARY INFORMATION

Distinct Structural Features of *Pseudomonas aeruginosa* ATP Synthase Revealed by Cryo Electron Microscopy

Meghna Sobti, Adam P. Gunn, Simon H. J. Brown, Lauren Zavan, Vesper M. Fraunfelter, Amanda L. Wolfe, Christopher A. McDevitt, P. Ryan Steed and Alastair G. Stewart.

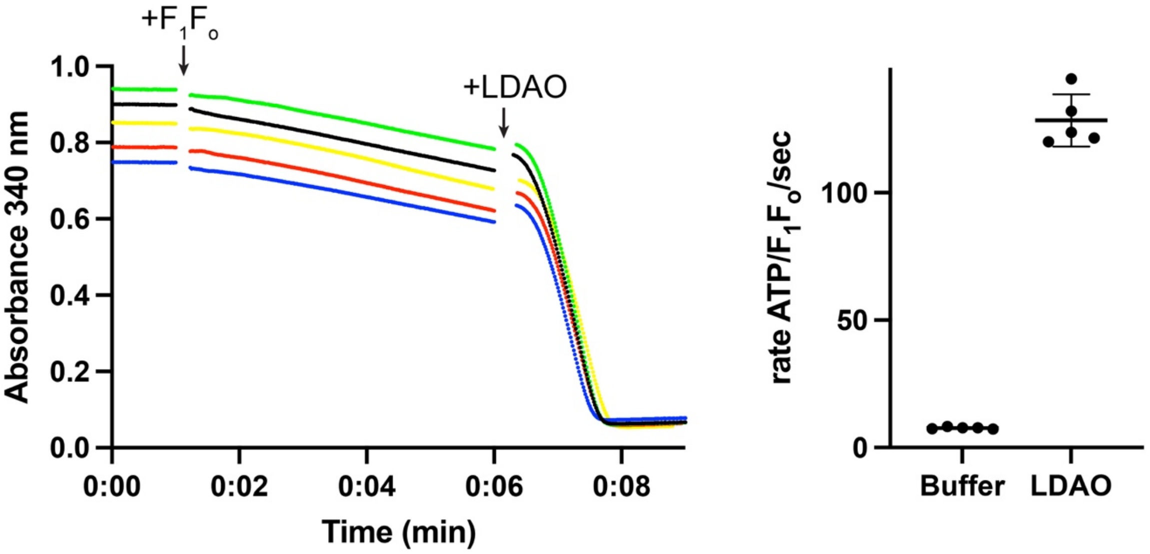

Figure S1: ATP regeneration assays of purified *P. aeruginosa* ATP synthase. Regeneration assays performed as detailed in methods. "+F_1F_o" arrow indicates addition of 8.4 µg *P. aeruginosa* ATP synthase and "+LDAO" arrow indicates addition of LDAO. Rates were calculated from the maximum slope over a 5 s rolling window using the NADH extinction coefficient and expressed as ATP molecules per F_1F_o complex per second. In the absence of LDAO, the mean rate was 7.6 ± 0.4 ATP/F_1F_o/sec; after LDAO addition, the rate increased ~17-fold to 128.4 ± 10.2 ATP/F_1F_o/sec (mean ± SD, n = 5, non-biological replicates). Absorbance spikes caused by opening/closing the spectrophotometer lid were omitted for clarity.

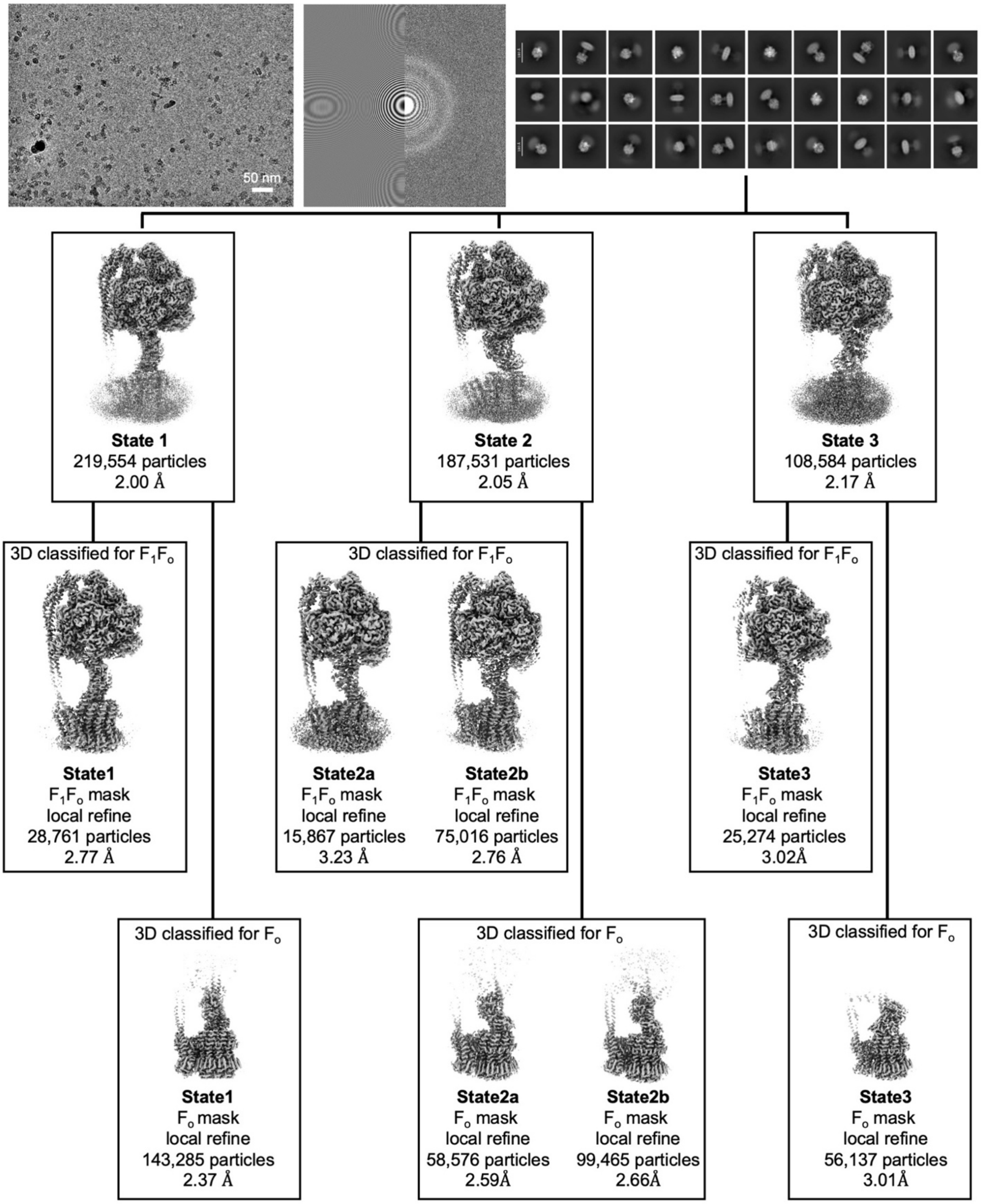

Figure S2: *P. aeruginosa* ATP synthase +10 mM MgADP cryo-EM data processing summary. Electron micrograph, with 50 nm scale bar, with corresponding power spectrum. Particles were picked and subjected to 2D classification. Particles were sorted into three classes using heterorefine, that represent three F_1 rotary states. Particles were then 3D classified using a mask of the F_o region. Particles were grouped based on the strength of density to make either F_1F_o focused maps or F_o focused maps.

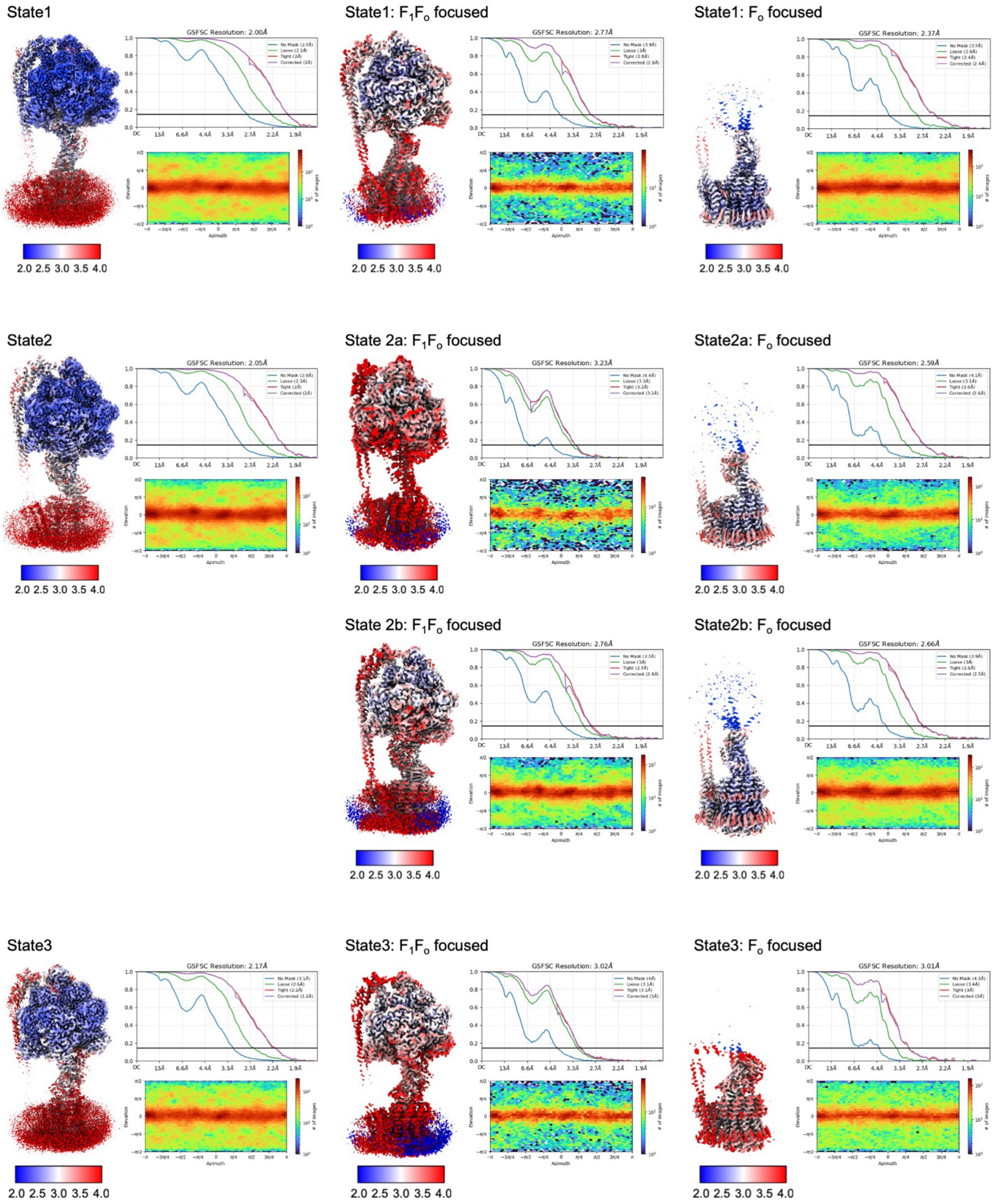

Figure S3: *P. aeruginosa* ATP synthase +10 mM MgADP cryo-EM local resolution, FSC and angular distribution. Cryo-EM information from cryoSPARC.

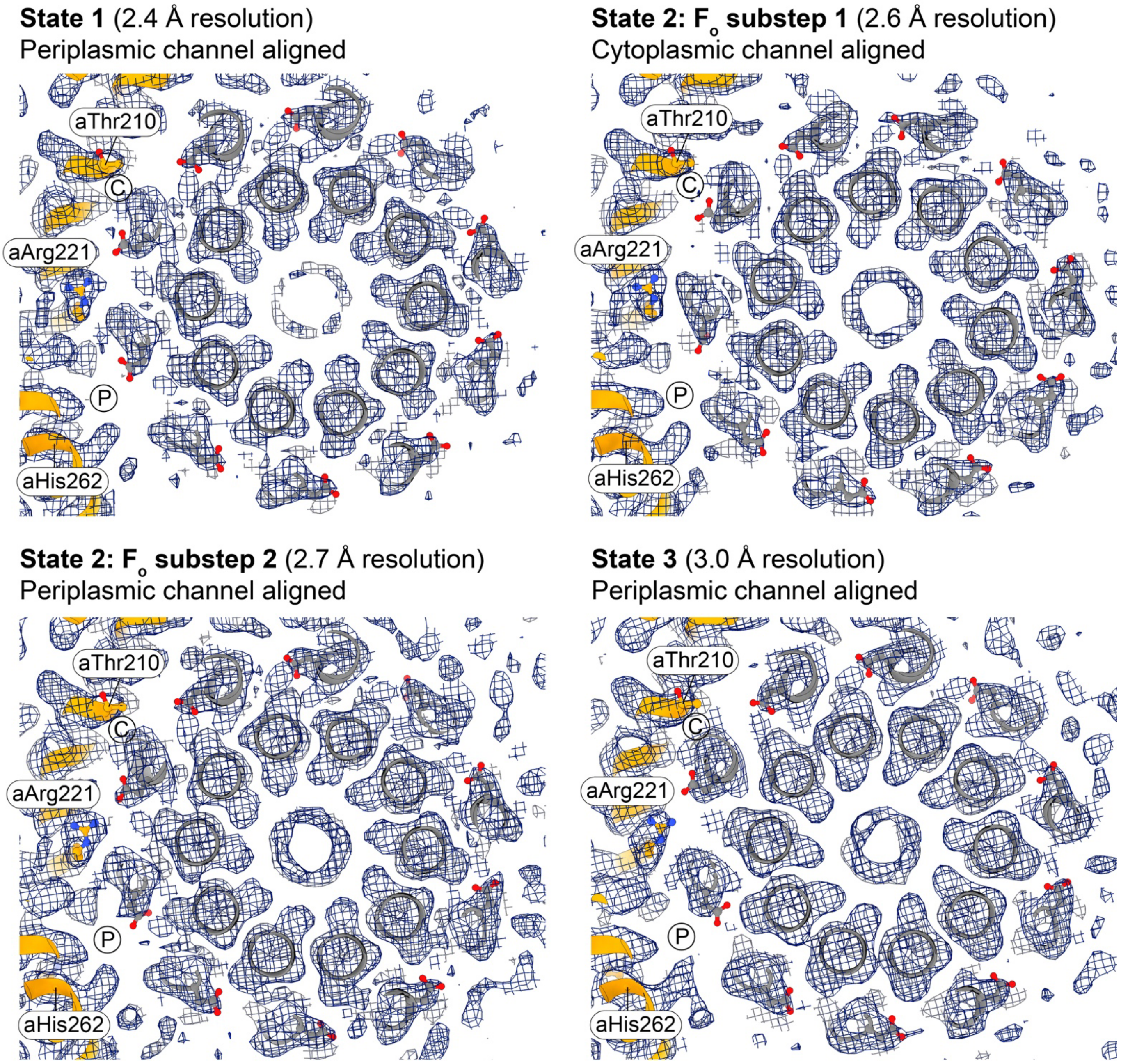

Figure S4: Four maps of the F_o motor. Masked 3D classification and focused refinement on the F_o region of the *P. aeruginosa* ATP synthase +10 mM MgADP dataset yielded four distinct maps; State 1, State 2: F_o substep 1, State 2: F_o substep 2, and State 3. In State 1, State 2 F_o substep 2 and State 3 the c-ring adopts the rotary sub-state, positioning the carboxylate of cAsp61 toward the periplasmic half-channel ((P) adjacent to aHis262). In State 2 substep 1, the c-ring is rotated ~11° relative to State 2 substep 2, bringing cAsp61 into alignment with the cytoplasmic half-channel ((C) adjacent to aThr210).

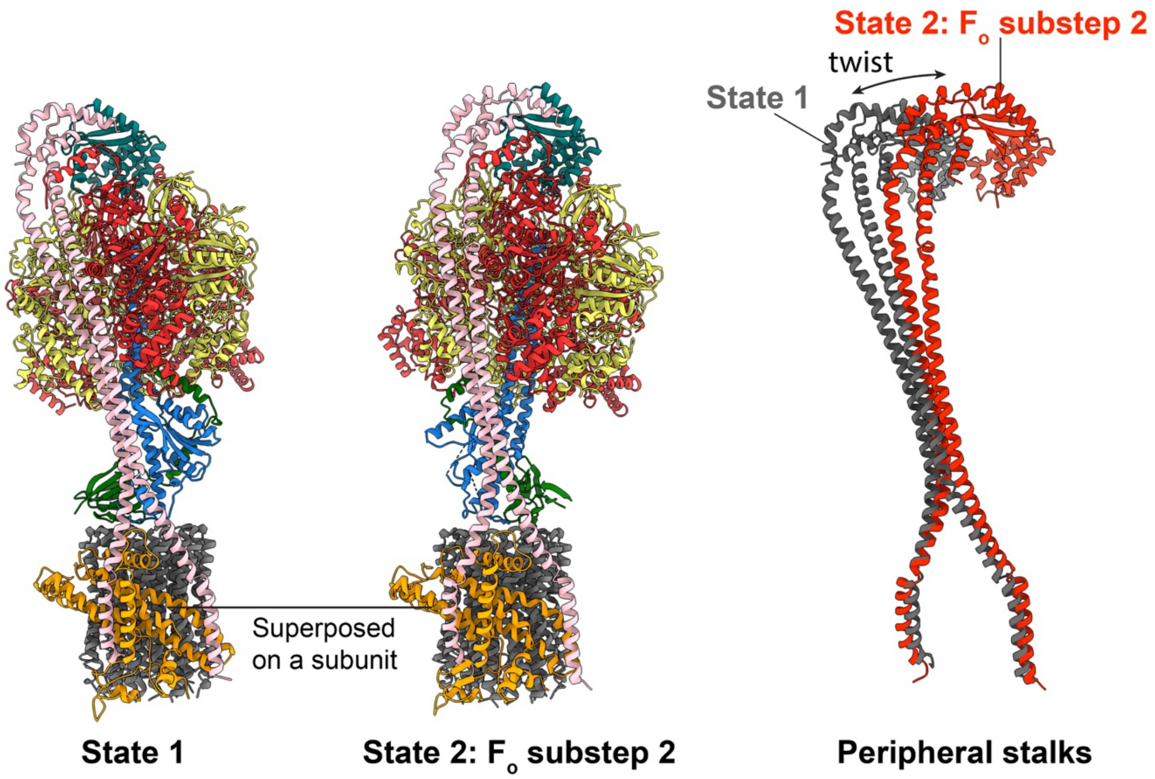

Figure S5: The peripheral stalk twists between rotary states. When states are superposed onto the a subunit, a tilting of F_1 relative to F_o can be observed. The peripheral stalks twist to accommodate this movement.

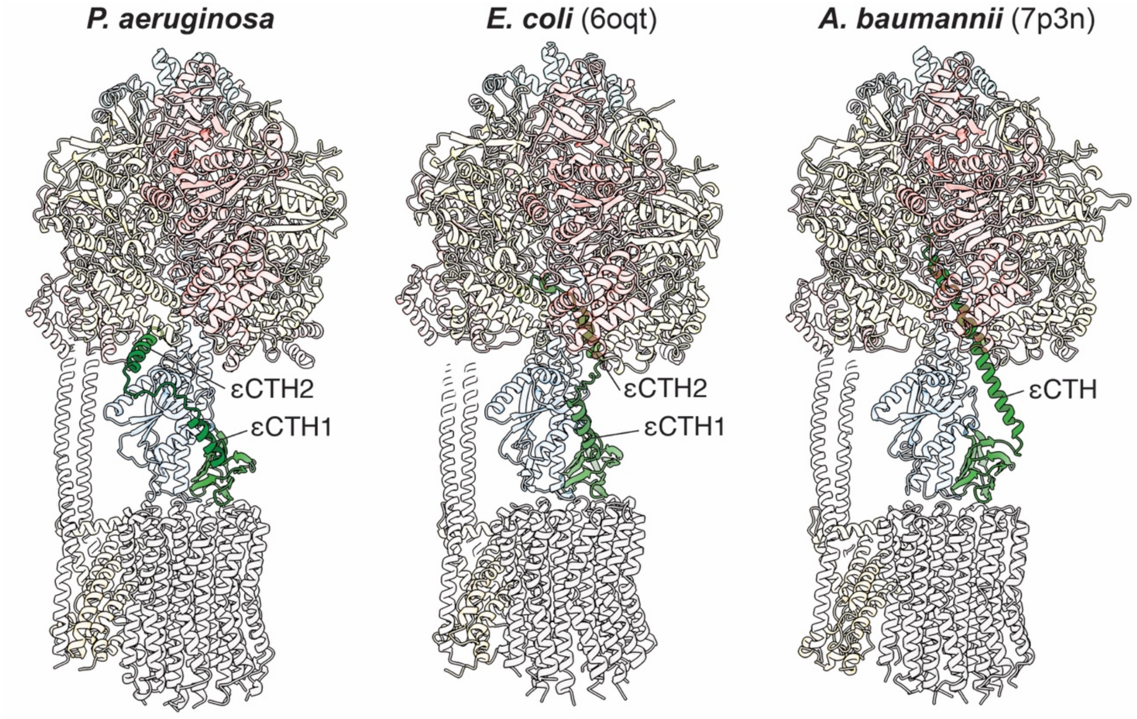

Figure S6: Comparison of ε C-terminal domains. εCTD of *P. aeruginosa* ATP synthase binds a different region of the F_1-ATPase in the extended "up" conformation compared to *E. coli* (PDB: 6oqt) and *A. baumannii* (PDB: 7p3n).

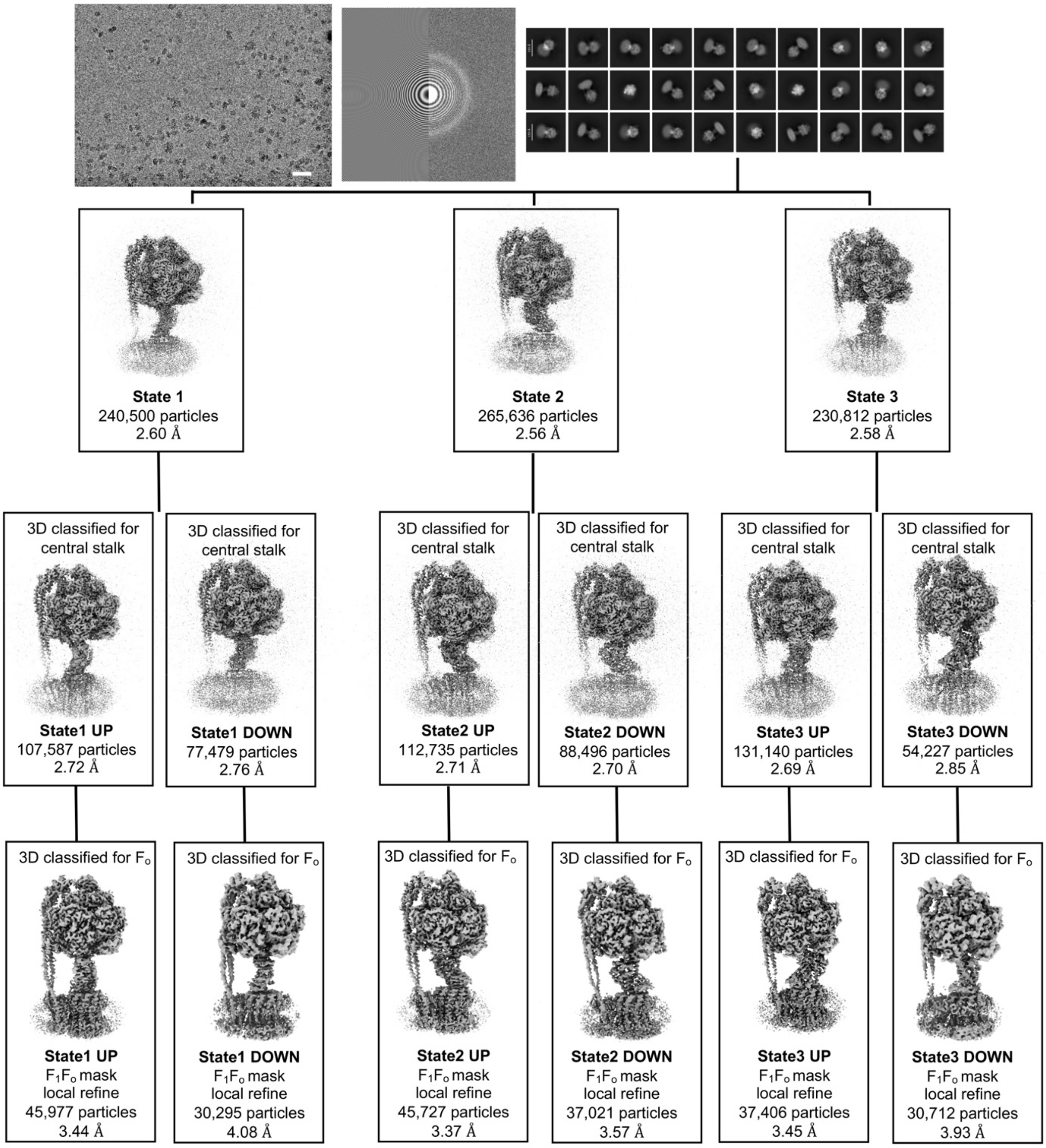

Figure S7: ***P. aeruginosa*** **ATP synthase +10 mM MgATP cryo-EM data processing summary.** Electron micrograph, with 50 nm white scale bar, with corresponding power spectrum. Particles were picked and subjected to 2D classification. Particles were sorted into three classes using heterorefine, that represent three F_1 rotary states. Particles were then 3D classified using a mask of the central stalk region. Particles were then 3D classified using a mask of the F_o region. Particles were grouped based on the strength of density and local refined using a mask of the F_1F_o (full enzyme).

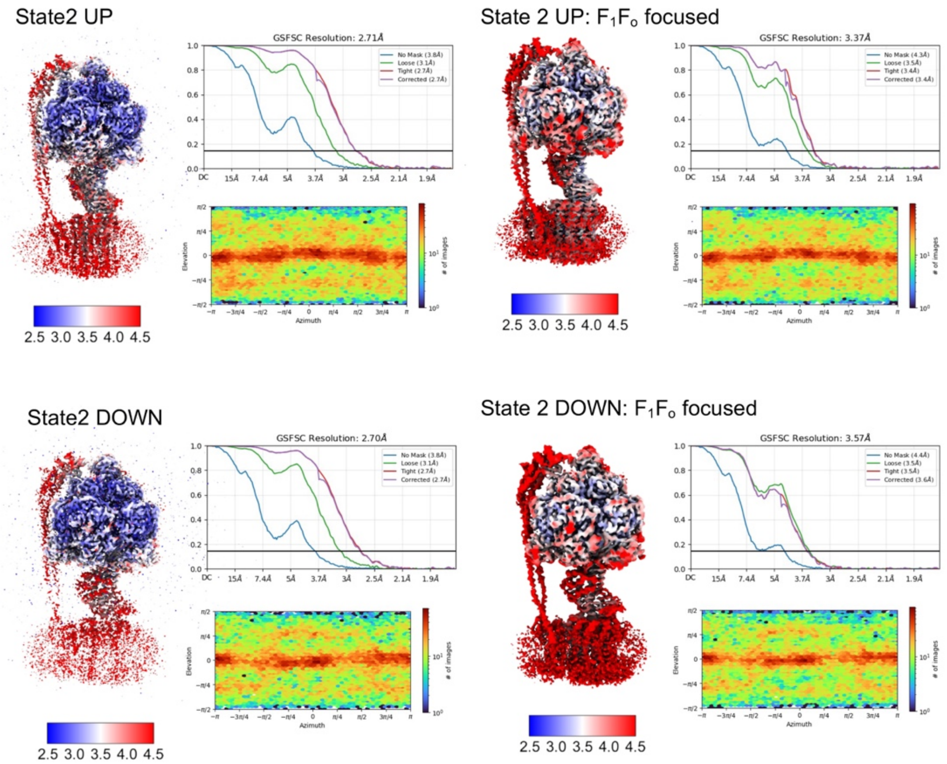

Figure S8: *P. aeruginosa* ATP synthase +10 mM MgATP cryo-EM local resolution, FSC and angular distribution. Cryo-EM information from cryoSPARC.

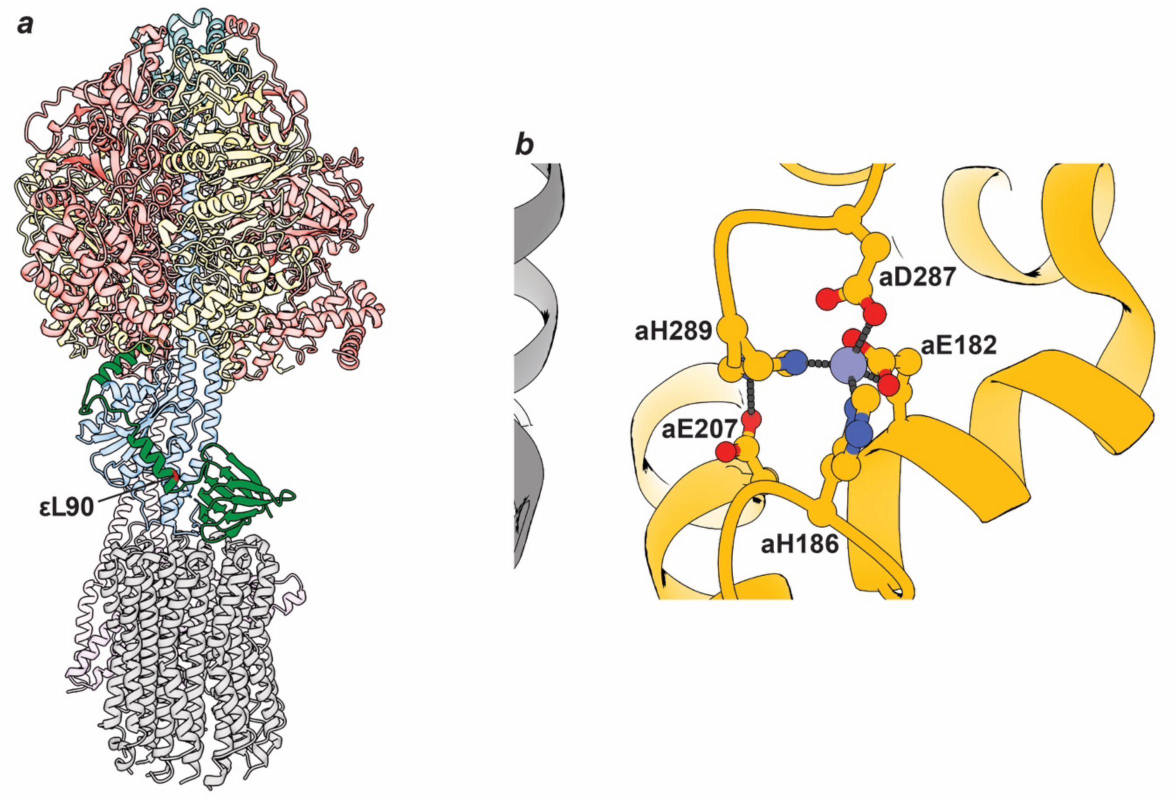

Figure S9: Mutant locations. Structural figure highlighting the location of mutations made in this study. (**a**) The ε90Δ mutant was created by truncation after Leu90 of subunit ε (residue colored red and labelled) to remove the εCTD. (**b**) The potential metal ion is coordinated by residues aGlu182, aHis186, aAsp287 and aHis289, with aGlu207 contacting aH289. aEC-like mutants were generated by replacing these residues with the structurally equivalent *E. coli* ATP AtpB residues: aHis186Gln, aAsp287Glu and aAsn288Δ.

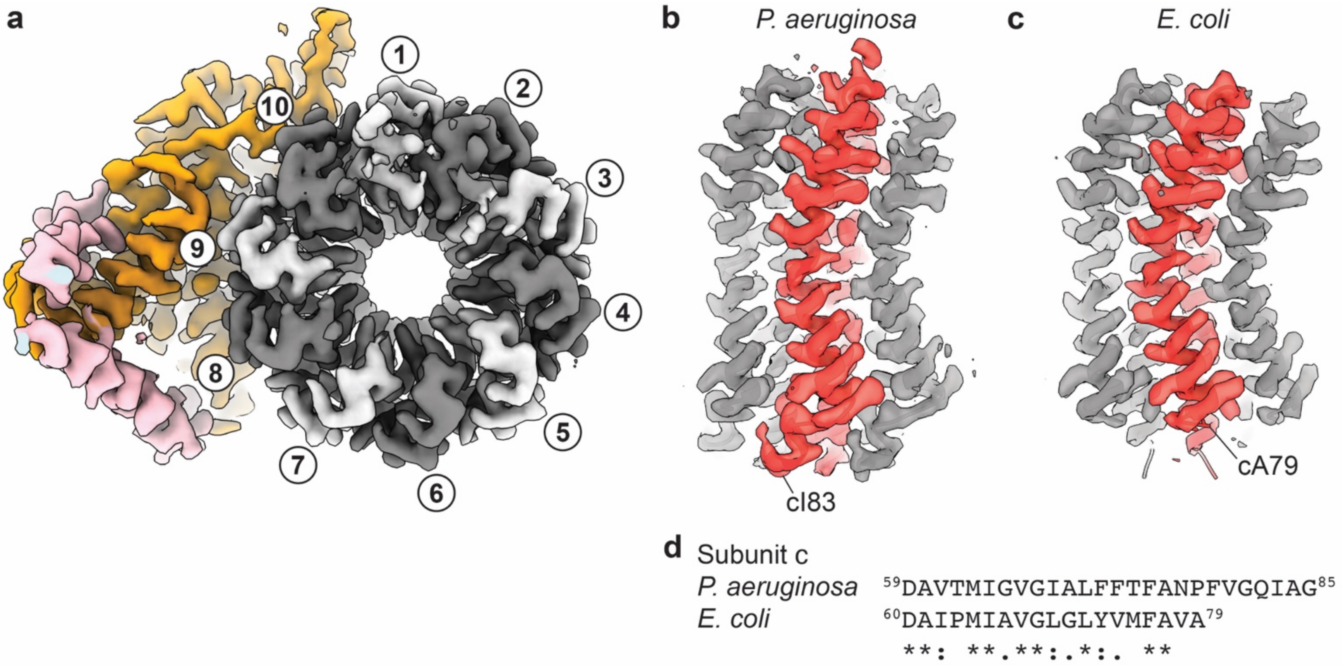

Figure S10: The c-ring of *P. aeruginosa* ATP synthase. (**a**) *P. aeruginosa* ATP synthase has ten c subunits in the F_o c-ring (State 1 F_o focused map). c subunits colored alternating grey and light grey and numbered to aid depiction. (**b-d**) The C-termini of *P. aeruginosa* ATP synthase subunit c contains a small extension compared to *E. coli*. Extracted map of three c subunits, with one c subunit colored red to allow for comparison. *P. aeruginosa* ATP synthase State 1 F_o focused map/model. *E. coli* ATP synthase EMD: 21419 and PDB: 6VWK. Sequence alignment of the C-terminal region of *P. aeruginosa* PA14 and *E. coli* K12 ATP synthase shows a seven amino acid extension.

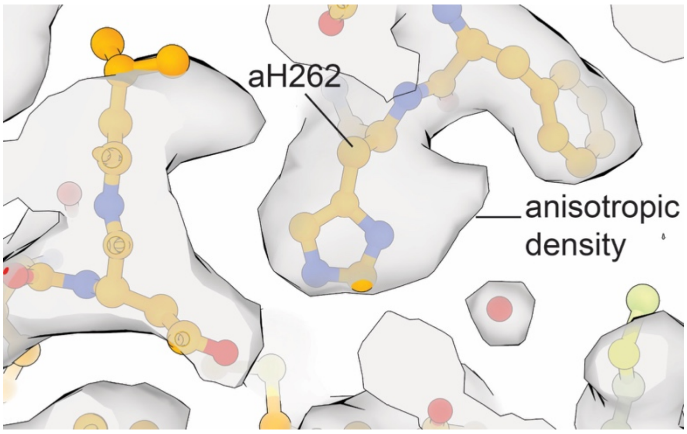

Figure S11: Cryo-EM map around aH262 of subunit a. Close up view of *P. aeruginosa* ATP PA14 synthase State 1 F_o focused map shows anisotropic density for aH262a.

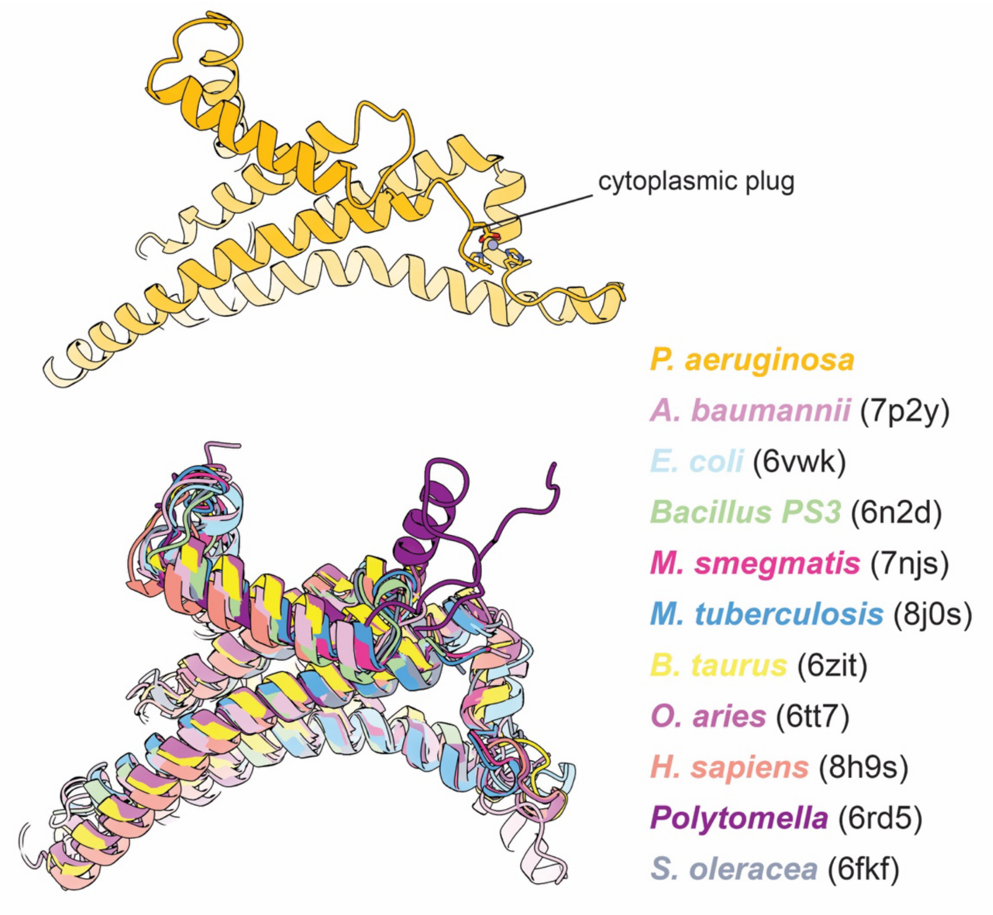

Figure S12: Structural comparison of subunit a. Comparison with other known structures of ATP synthase subunit a (colored and labelled with PDB ID in brackets, species shown in Table S2). The unique closed cytoplasmic channel, shown by the black line as the 'cytoplasmic plug', and coordinated metal ion, residues shown as sticks and the metal ion as a grey sphere, in the *P. aeruginosa* PA14 ATP synthase are highlighted.

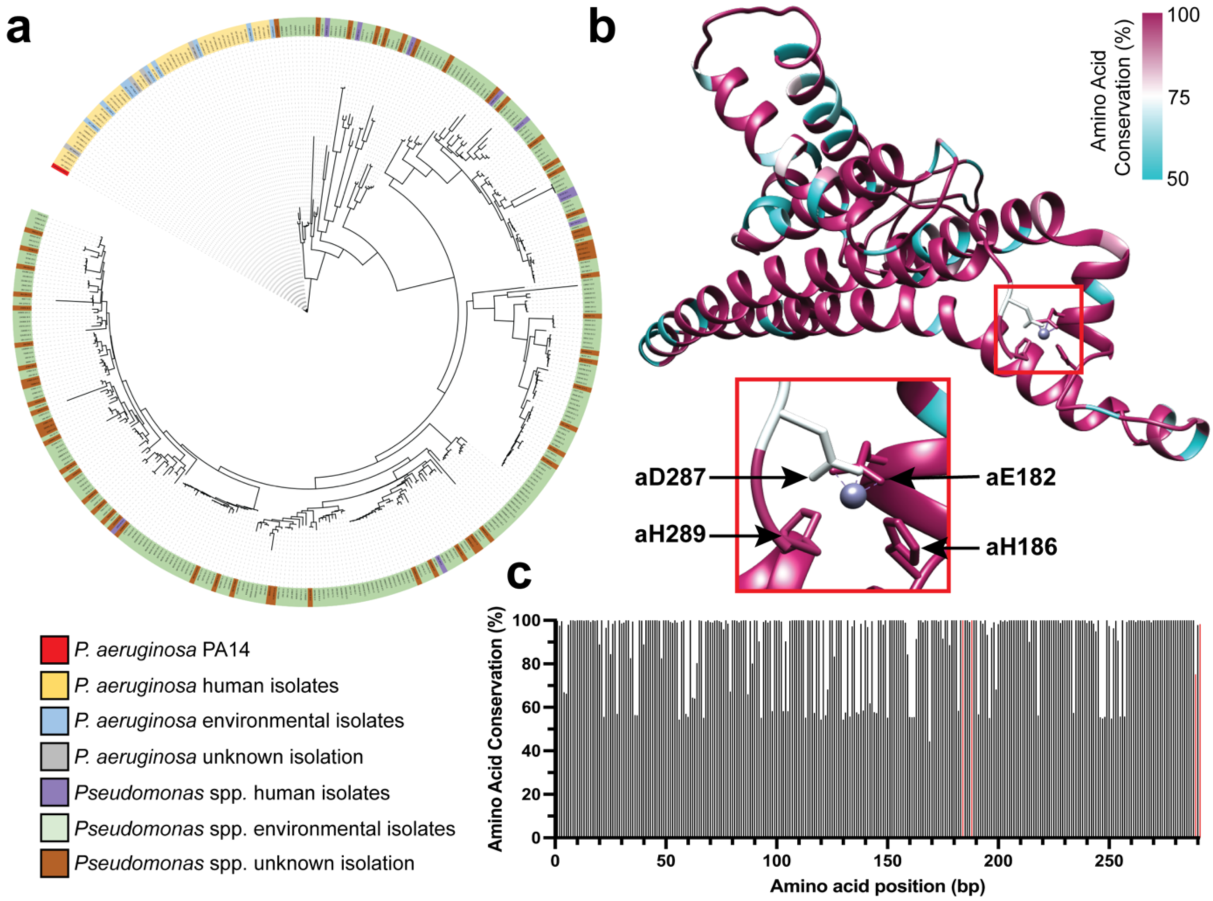

Figure S13: Conservation of AtpB in *Pseudomonas* species. (**a**) Phylogenetic analysis of 384 unique *atpB* nucleotide sequences from 1,042 publicly available *Pseudomonas* spp. genomes that contained *atpB*. Environmental and human isolates are as indicated and PA14 (reference strain) is indicated in red. The translated AtpB sequences from the 1,042 publicly available *Pseudomonas* spp. genomes were used to: (**b**) map conservation to the tertiary structure of *P. aeruginosa* zinc-bound AtpB. Metal coordinating residues are indicated by black arrows and zinc ion is in purple; and (**c**) determine the frequency of amino acid conservation at each position within the protein. Metal-coordinating residues are indicated by red bars.

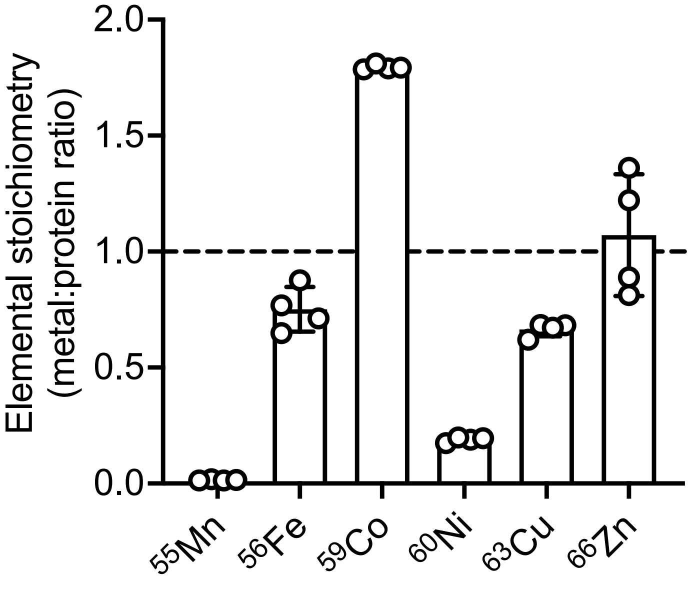

Figure S14: Elemental analysis of purified recombinant *P. aeruginosa* F_1F_o ATP synthase. Molar ratios were calculated from the concentration of each element determined by ICP-MS analysis performed on the detergent-soluble *P. aeruginosa* ATP synthase protein complex. Data shown are the mean ± standard deviation of four independent analyses with individual values shown by circles. n=4.

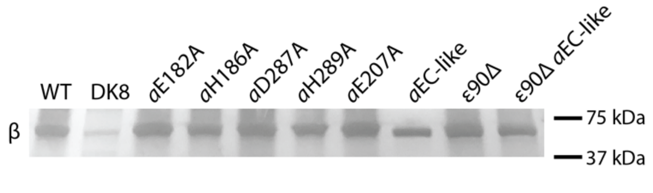

Figure S15: Expression and assembly of *P. aeruginosa* ATP synthase is not affected by the selected mutations. To determine whether similar amounts of *P. aeruginosa* ATP synthase were present in inverted vesicles prepared from the pASH20 mutants, immunostaining using a species cross-reactive antibody against subunit β was performed. Wild-type (WT) (pASH20) and untransformed DK8 were run as positive and negative control, respectively. A prominent band consistent with the expected size of subunit β (50 kDa) was visible at approximately equal intensity for all mutants suggesting that none of the subunit *a* mutations or truncation of subunit ε significantly affects expression or assembly of F_1F_o.

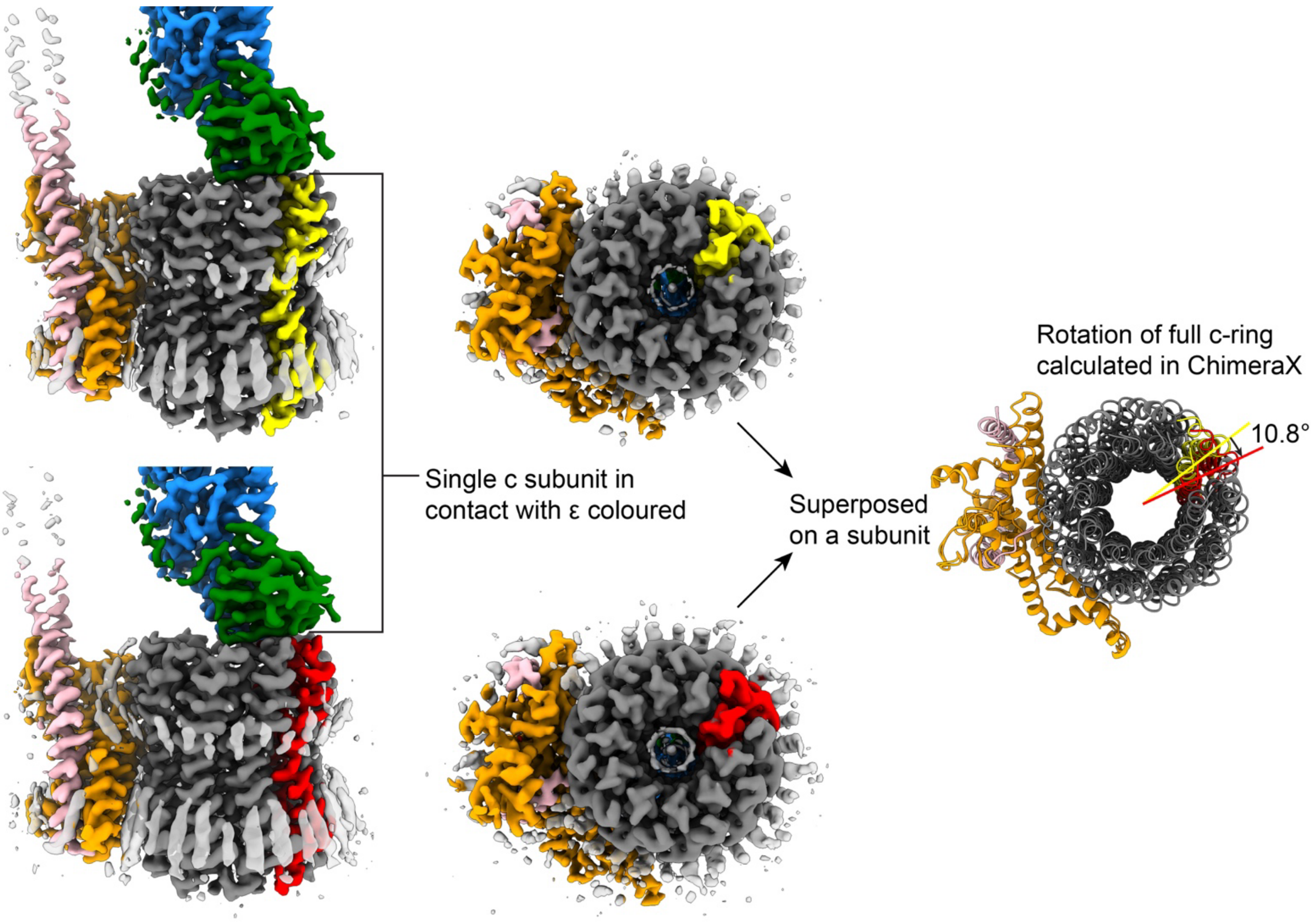

Figure S16: Quantification of c-ring rotation between F_o substeps. To measure the ~11° rotation of the c-ring between State 2 F_o substep 1 and State 2 substep 2, we used ChimeraX[64] as follows. First, we selected the same c subunit that contacts the ε subunit in both models as a marker (map colored yellow and red). Each structure was then superposed on subunit a using the "matchmaker" command. Then the relative rotation of the c-ring vs the a subunit was determined with the "measure rotation" command. The rotation (black arrow) is indicated with red and yellow lines showing the same c subunit rotary position in each state.

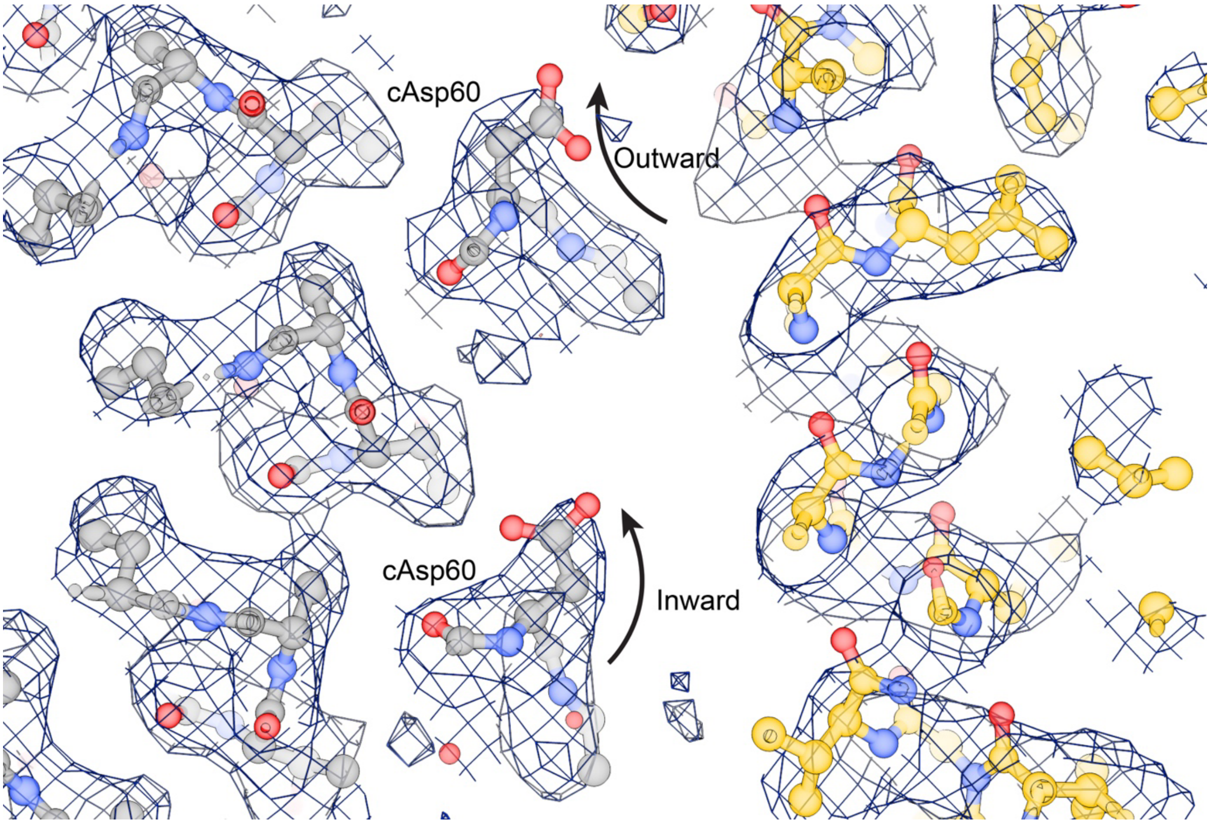

Figure S17: Alternative rotameric states of cAsp60. A cross-sectional slice through the State 1 F_o focused cryo-EM map highlights two adjacent cAsp60 residues (shown as ball and sticks) adopting different side-chain conformations. One rotamer points inward toward the c-ring axis, whereas the other points outward away from the c-ring axis.

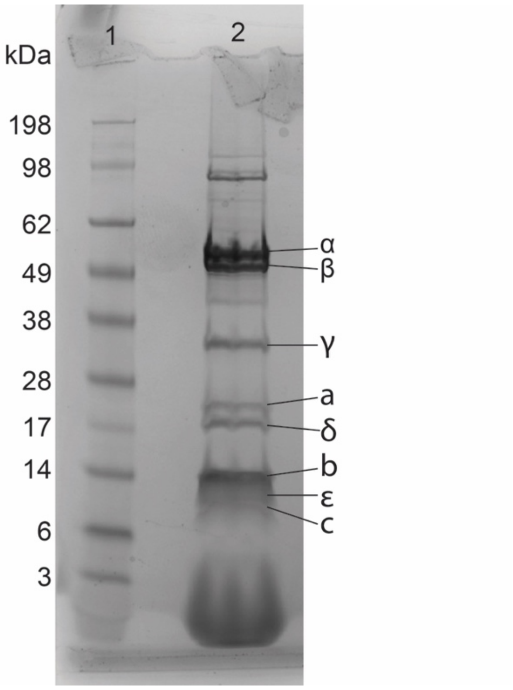

Figure S18: Purification gel. Coomassie stained SDS-PAGE of *P. aeruginosa* ATP synthase used in this study (recombinantly expressed in *E. coli* DK8 cells, purified in digitonin detergent). Lane 1 SeeBlue Plus 2 markers. Lane 2 purified protein subjected to cryo-EM.

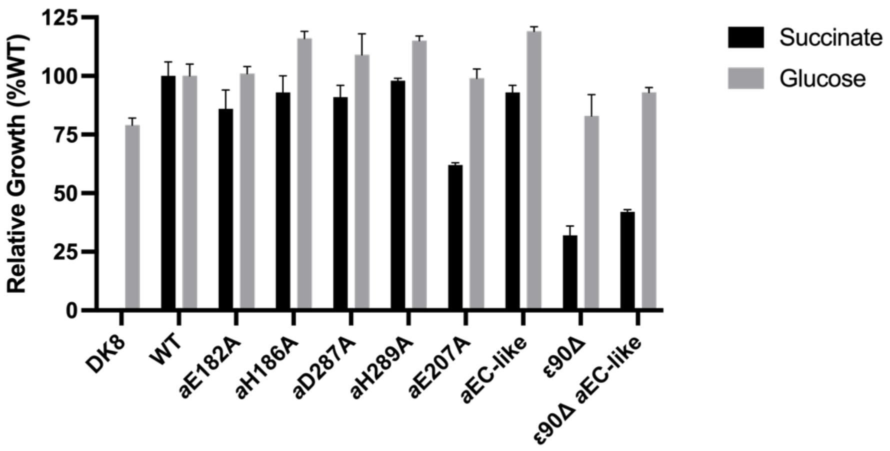

Figure S19: Growth of *P. aeruginosa* ATP synthase mutants on minimal medium. The ability of mutant *P. aeruginosa* ATP synthase expressed in *E. coli* DK8 to support growth on minimal medium was assayed. Transformant *E. coli* strains were grown on M63-TIV medium supplemented with either 0.6% succinate (black bars) or 0.04% glucose (gray bars), with growth was measured by optical density at 550 nm. Maximum growth after 6 h on glucose or 12 h on succinate medium was normalized to the growth of *E. coli* DK8 pASH20 (WT). Data represents the mean ± standard deviation of n=3 independent replicates. aE207A, ε90Δ and ε90Δ aEC-like show reduced growth on succinate medium relative to WT.

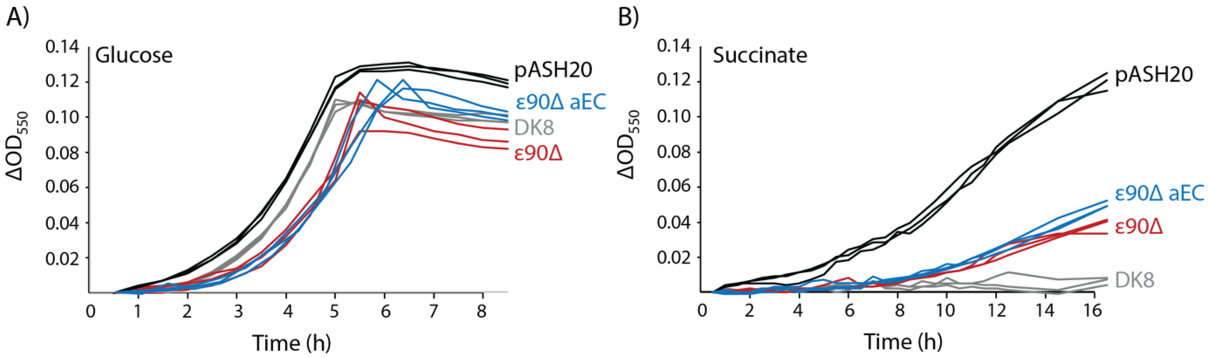

Figure S20: Growth curves of ε90Δ and ε90Δ aEC mutant in minimal medium. *E. coli* DK8 cells transformed with pASH20 (WT, black traces), pASH20 ε90Δ or pASH20 ε90Δ aEC-like were grown in minimal medium liquid broth with 0.04% glucose (**A**) or 0.6% succinate (**B**) as the sole carbon source. Growth was monitored by measuring the optical density at 550 nm (OD_{550}) at 30 min intervals for 16 h, and background (OD_{550} at t = 30 min) was subtracted. n=3, no statistics performed in this figure.

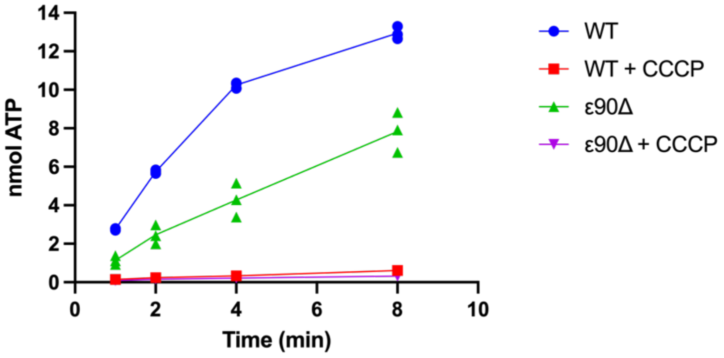

Figure S21: Sample *P. aeruginosa* ATP synthesis data. ATP synthesis activity of inverted inner membrane vesicles prepared from *E. coli* transformants expressing WT (*blue circles*) or ε90Δ (*green triangles*) *P. aeruginosa* ATP synthase was assayed using a luciferin/luciferase system (see Methods). Samples were removed from the reactions at *t* = 1, 2, 4, and 8 min into stop solution and diluted before mixing with luciferin/luciferase and measuring luminescence. Known concentrations of ATP were included on each assay plate, and the resulting standard curve was used to convert luminescence values nmol ATP in the synthesis reaction. Controls containing 0.2 mM CCCP were included for each mutant (*red squares* and *purple inverted triangles*, respectively) showing negligible gradient-independent ATP synthesis, which was subtracted as background. ATP synthesis rate was typically linear to at least 4 minutes, so quantity of ATP synthesized after 4 minutes was used to calculate ATP synthesis activities shown in Fig. 7. n=3 for WT and ε90Δ, n=1 for WT + CCCP ε90Δa + CCCP, no statistics performed in this figure.

Table S1: Conservation analysis of the *atpB* gene and metal coordinating residues in a *Pseudomonas* species database.

Counts	***atpB* gene**	**aE182**	**aH186**	**aD287**	**aH289**
Present	1042	1042	1042	783	1036
Absent	31	40	40	46	46
Stops	9	-	-	-	-
Residue change to E	-	-	-	252	-
Residue change to Q	-	-	-	1	-
Total *Pseudomonas* spp.	1082	1082	1082	1082	1082
Ratio present (%)	96.30%	96.30%	96.30%	72.37%	95.75%

Table S2: Representative AtpB metal binding site sequence analyses

**Organism**	**aE182***	**aH186**	**aD287**	**aN288**	**aH289**
P. aeruginosa PA14	E	H	D	N	H
P. aeruginosa PAO1	E	H	D	N	H
A. baumannii ATCC 17978	E	N	K	-	H
E. coli K12	E	Q	E	-	H
Geobacillus stearothermophilus PS3	R	R	H	D	H
Mycobacterium smegmatis mc^{2}155	K	G	L	D	H
Mycobacterium tuberculosis ATCC 25618	K	G	L	E	E
Saccharomyces cerevisiae W303	F	G	L	-	H
H. sapiens	F	G	-	-	-
Spinacia oleracea	G	Q	G	H	H
M. solifugus	D	-	V	Q	D

* Colors represent residue conservation or a conservative substitution (e.g. E → D and residue present or not at position a288).

Table S3: Cryo-EM data collection and refinement statistics for +10 mM MgADP.

	#1 State1 (EMDB-49999) (PDB 9O19)	#2 State1 F1Fo focused (EMDB-70000) (PDB 9O1A)	#3 State1 Fo focused (EMDB-70001) (PDB 9O1B)	#4 State2 (EMDB-70002) (PDB 9O1C)	#5 State2a F1Fo focused (EMDB-70003) (PDB 9O1D)	#6 State2a Fo focused (EMDB-70004) (PDB 9O1E)	#7 State2b F1Fo focused (EMDB-70005) (PDB 9O1F)	#8 State2b Fo focused (EMDB-70006) (PDB 9O1G)	#9 State3 (EMDB-70007) (PDB 9O1H)	#10 State3 F1Fo focused (EMDB-70009) (PDB 9O1J)	#11 State3 Fo focused (EMDB-70010) (PDB 9O1K)
**Data collection and processing**											
Magnification	59,000x	59,000x	59,000x	59,000x	59,000x	59,000x	59,000x	59,000x	59,000x	59,000x	59,000x
Voltage (kV)	300	300	300	300	300	300	300	300	300	300	300
Electron exposure (e–/Å^2)	62	62	62	62	62	62	62	62	62	62	62
Defocus range (μm)	0.5-1.5	0.5-1.5	0.5-1.5	0.5-1.5	0.5-1.5	0.5-1.5	0.5-1.5	0.5-1.5	0.5-1.5	0.5-1.5	0.5-1.5
Pixel size (Å)	0.83	0.83	0.83	0.83	0.83	0.83	0.83	0.83	0.83	0.83	0.83
Symmetry imposed	C1	C1	C1	C1	C1	C1	C1	C1	C1	C1	C1
Initial particle images (no.)	770,176	219,553	219,554	770,176	187,531	187,531	187,531	187,531	770,176	108,584	108,584
Final particle images (no.)	219,554	28,761	143,285	187,531	15,867	58,576	75,016	98,465	108,584	25,274	56,137
Map resolution (Å)	2.00	2.77	2.37	2.05	3.23	2.59	2.76	2.66	2.17	3.02	3.01
FSC threshold 0.143											
**Refinement**											
Initial model used (PDB code)	Model Angelo	Model Angelo	Model Angelo	Model Angelo	Model Angelo	Model Angelo	Model Angelo	Model Angelo	Model Angelo	Model Angelo	Model Angelo
Model resolution (Å)	2.0	2.7	2.4	2.0	3.2	2.6	2.7	2.6	2.2	3.0	3.0
FSC threshold 0.143											
Map sharpening *B* factor (Å^2)	-35.4	-42.4	-43.7	-35.0	-33.0	-42.3	-56.2	-52.6	-33.7	-39.3	-59.1
Model composition											
Non-hydrogen atoms	30,261	37,097	8,956	28,855	37,036	8,954	37,043	8,960	28,781	36,965	9,114
Protein residues	3,788	4,903	1,213	3,780	4,895	1,213	4,895	1,213	3,771	4,886	1,233
Ligands	12	13	1	12	12	1	12	1	12	12	
B factors (Å^2)											
Protein	36.93	26.64	33.76	25.84	26.92	33.76	26.93	33.76	25.54	26.74	32.95
Ligand	23.24	48.65	30.00	48.35	48.75	30.00	48.13	30.00	48.75	48.75	
R.m.s. deviations											
Bond lengths (Å)	0.009	0.012	0.002	0.012	0.012	0.003	0.012	0.003	0.013	0.012	0.011
Bond angles (°)	1.395	1.958	0.580	1.988	2.004	0.619	2.018	0.646	1.956	1.955	19.59
Validation											
MolProbity score	0.80	0.76	1.26	0.91	0.74	1.15	0.89	1.41	1.14	1.09	0.81
Clashscore	1.03	0.67	4.5	0.85	0.39	2.93	0.96	3.36	1.97	1.94	1.07
Poor rotamers (%)	0.7	0.73	1.10	0.13	0.31	1.22	0.26	2.32	0.2	0.32	0.33
Ramachandran plot											
Favored (%)	98.41	97.80	98.65	97.18	97.50	98.23	97.50	98.82	96.98	97.31	98.59
Allowed (%)	1.59	2.16	1.35	2.77	2.46	1.77	2.46	1.18	2.99	2.67	1.41
Disallowed (%)	0.00	0.04	0.00	0.05	0.04	0.00	0.04	0.00	0.03	0.02	0.00

Table S4: Cryo-EM data collection and refinement statistics for +10 mM MgATP.

	#12<br>State2 with 10mM ATP F1Fo focused “Up”<br>(EMDB-71967)	#13<br>State2 with 10mM ATP F1Fo focused “Down”<br>(EMDB-71968)
**Data collection and processing**		
Magnification	59,000x	59,000x
Voltage (kV)	300	300
Electron exposure (e–/Å^2)	74	74
Defocus range (μm)	0.5-1.5	0.5-1.5
Pixel size (Å)	0.83	0.83
Symmetry imposed	C1	C1
Initial particle images (no.)	265,636	265,636
Final particle images (no.)	45,727	37,021
Map resolution (Å)<br>FSC threshold 0.143	3.37	3.57
